# Omission of lateral isocenter shifts in robust optimization of proton therapy treatment plans for prostate cancer

**DOI:** 10.1002/acm2.70833

**Published:** 2026-10-14

**Authors:** Fionnbarr T O'Grady, Jackeline Castro, Daniel W Kim, Jiajin Fan, Peng Wang

**Affiliations:** ^1^ Inova Schar Cancer Institute Inova Health System Fairfax Virginia USA

**Keywords:** optimization, planning time, prostate, Protons, robustness

## Abstract

**Background:**

Proton therapy is routinely used to treat prostate cancer using bilaterally opposed beams and can achieve lower dose to nearby organs‐at‐risk (OARs) such as the bladder and rectum. Pencil beam scanning (PBS) proton therapy offers the most conformal treatment plans to best spare healthy tissue. Robust optimization is often utilized in planning PBS treatment to account for uncertainties in treatment delivery, such as misalignment of the isocenter, by evaluating the dose in perturbation scenarios in which the isocenter is translated. This process is effective but resource intensive and time consuming.

**Purpose:**

To assess the feasibility of omitting lateral translations of the isocenter in robust optimization of pencil‐beam scanning (PBS) proton therapy treatment plans for prostate cancer patients, utilizing parallel opposed lateral beams.

**Methods:**

The impact of isocenter shifts in the beam direction was studied systematically in a series of cube plans of varying field size and depth in water. In addition, the impact of omitting lateral isocenter shifts in robust optimization was analyzed retrospectively across sixteen clinical treatment plans, including plans with pelvic lymph nodes (LN) and a simultaneous integrated boost (SIB) delivered to the dominant intraprostatic lesion (DIL) or PET avid LNs. The impact on key dose‐volume metrics for several OARs, as well as the results of robust evaluation and the difference in optimization time were recorded.

**Results:**

The plan comparison shows no significant difference in dose to any OAR or robust target coverage for any of the plans considered. The time required for a single optimization with fixed settings reduced by 24 min (22%) on average, with a maximum reduction of 129 min (43.1%).

**Conclusions:**

Lateral isocenter shifts can be safely omitted in robust optimization of PBS proton therapy treatment plans for prostate patients, utilizing parallel opposed lateral beams, to achieve a significant reduction in optimization time, without increasing dose to OARs or sacrificing robust target coverage.

## 1 INTRODUCTION

While the clinical benefit of proton beam therapy (PBT) over conventional radiation therapy in the treatment of prostate cancer is still debated,^1^, ^2^ many studies have demonstrated a clear dosimetric benefit in terms of reduced dose to organs‐at‐risk (OARs) such as the bladder and rectum.^3^, ^4^, ^5^ More extensive level one data is needed to determine if this dosimetric benefit translates to improved patient outcomes in the form of increased local control and/or reduced normal tissue toxicity. Several randomized controlled clinical trials, such as PARTIQoL, PAROS and COMPPARE, are under way that may provide more evidence to help answer this question.^6^, ^7^, ^8^ Although the initial results of PARTIQoL, at a median follow‐up of 60.3 months, have shown no difference between PBT and Intensity modulated radiation therapy (IMRT) in terms of progression‐free survival or patient reported toxicities, outcomes will continue to be monitored at longer follow up time and for additional endpoints. Despite this, the dosimetric benefit alone has allowed PBT to become common in the treatment of prostate cancer in the United States. A 2022 JAMA Network Open study found that prostate cancer, along with breast cancer, were the most common cancers treated with PBT in the US between 2004 and 2018.^9^ At our institution pelvic patients, of which the majority are prostate, make up 22.9% of our total volume of patients treated with PBT.

The current state of the art in PBT is pencil beam scanning (PBS), in which a narrow proton beam is scanned throughout the target using a combination of varying beam energy and fast scanning magnets to deliver the prescribed dose to the target while minimizing dose to healthy tissue. PBS represents a substantial improvement in conformality compared to passive scattering techniques, specifically on the proximal edge of the target.^10^, ^11^, ^12^, ^13^ In addition, it allows for intensity modulated proton therapy (IMPT), implemented via multi‐field optimization (MFO), which can reduce dose to normal tissue even further by allowing different portions of the target to be covered by different subsets of beams in the treatment plan.^14^, ^15^ This is in contrast to single field optimization (SFO) in which all beams cover the target with an equal uniform dose. Determining the proton spot map needed to produce the desired dose distribution requires inverse planning techniques, in which a cost function, defined by a set of optimization objectives, is minimized. The sensitivity of proton dose distributions to uncertainties means that the standard PTV approach to ensuring robust target coverage is inadequate.^16^, ^17^ A common approach used in PBT is robust optimization (RO), in which uncertainties are incorporated directly into the optimization process by minimizing the cost function across a range of perturbation scenarios. The standard of practice in the field is to include perturbations for beam range error, deriving from inaccurate estimation of proton stopping power ratios (SPRs), and patient alignment error in three dimensions.^18^, ^19^ In our institution the uncertainties for prostate treatment are range error = ± 3.5% and patient alignment error = ± 0.5 cm, which leads to 3 range error scenarios (0 ± 3.5%) multiplied by 7 misalignment scenarios (0, ± 0.5 cm x, ± 0.5 cm y, ± 0.5 cm z) for a total of 21 RO scenarios. RO is followed by robust evaluation (RE) in which the final plan is forward calculated in a series of perturbation scenarios to ensure that adequate robustness has been achieved according to a set of institutional criteria. The coverage criteria for RE is V95% > 95% which must be met for all CTVs in all perturbation scenarios while the OAR goals must be met in at least 50% of scenarios. RO is effective but time consuming, especially for Monte Carlo (MC) optimization in which the dose in each iteration is calculated with a MC dose engine. The optimization time increases with the number of perturbation scenarios specified by the planner.

It is important to reduce optimization time to the extent possible to minimize the computing and human resources required for each plan and to avoid unnecessary delays in treatment. Reducing the time and resources needed per plan can lead to an increase in patient throughput, which is especially important in the context of PBT, given its limited availability. Alternatively, it can allow resources to be utilized for other purposes such as enhanced quality assurance or clinical development projects that improve efficiency and patient safety over time.^20^

The standard beam arrangement for prostate cancer is a pair of opposed lateral beams. For cases involving the prostate only or the prostate plus the proximal seminal vesicles an SFO technique is utilized. In cases involving lymph nodes (LNs) a hybrid approach is taken in which most of the target, including the prostate and the medial LNs are planned SFO while the lateral LNs are planned MFO in which each side is covered by the ipsilateral beam only to avoid crossing the medial normal tissue with the contralateral beams.^21^ A posterior‐anterior (PA) beam may also be added in this case to avoid covering the lateral LNs with a single beam only. This improves robustness but may increase dose to the rectum. Some patients are treated with a simultaneous integrated boost (SIB) in which a higher dose is delivered to a dominant intraprostatic lesion (DIL) or to PET avid LNs.^22^, ^23^ For patients with hip prostheses the beam arrangement must be modified to avoid traversing the prosthesis. In this case the lateral beam on the side of the prosthesis is replaced by one or more oblique beams.^24^, ^25^

The primary factor affecting the dose delivered to a particular point within a patient by a proton beam is the water equivalent thickness (WET) in the beam path. The position of the planned isocenter relative to the actual radiation isocenter in the beam direction has minimal impact of the dose distribution. There is some impact caused by beam divergence, but it is negligible for isocenter deviations arising from patient setup error in clinical practice, given that the virtual source‐to‐axis distance (vSAD) is on the order of meters while setup error is on the order of millimeters. Furthermore, it primarily affects the dose profile in the perpendicular direction and leaves the profile along the beam axis largely unaffected. Isocenter deviations perpendicular, or partially perpendicular, to the beam axis can have a more significant impact as they can dramatically change the WET in the beam path. However, deviations entirely parallel to the beam axis should have negligible impact. Despite this it is common practice in many clinics to include lateral isocenter deviations in the perturbation scenarios used in RO of prostate plans. Table 1 summarizes the results of a cross‐institutional survey carried out in 2024–2025 in the USA showing that this practice is routine in several centers included in the survey. In principle, these deviations could be omitted from RO of prostate plans containing only lateral beams, which would reduce the number of RO scenarios from 21 to 15 and decrease optimization time. To justify this change in clinical practice it is necessary to estimate the reduction in optimization time and to demonstrate that it can be achieved without any degradation in plan quality, in particular robustness of target coverage.

**TABLE 1:** Survey results: Practice for optimization of PBS proton therapy plans for prostate patients across several institutions.

Institution	Proton System	TPS	Practice
1	IBA Proteus Plus	Eclipse	No robust optimization/evaluation, PBSTV used instead, No MC optimization
2	Varian ProBeam	Eclipse	RL shifts included in robust optimization, No MC optimization
3	IBA Proteus Plus	RayStation	RL shifts included in robust optimization, No MC optimization
4	IBA Proteus Plus	RayStation	RL shifts included in robust optimization, No MC optimization
5	Varian ProBeam	RayStation	lateral shift included in robust MC optimization
6	Mevion S250i	RayStation	lateral shift included in robust MC optimization
7	IBA Proteus One	RayStation	lateral shift included in robust MC optimization

## 2 METHODS

The study was carried out with the RayStation v11a TPS (Raysearch Laboratories, Stockholm, Sweden), utilizing a beam model for an IBA Proteus Plus PBS proton therapy system. In our clinic RayStation is hosted on servers with 24 virtual CPU cores and 265GB of RAM per server. The physical CPUs are 2x AMD 75F3 and each server is equipped with a NVidia A40 GPU. RayStation v11a utilizes both CPU and GPU in combination for both optimization and dose calculation.

First the impact of isocenter deviations parallel to the beam axis was measured in a series of cube plans of varying depth and field size generated in water. The cube plans were calculated with a dose grid resolution of 0.1 cm and a MC uncertainty of 0.1%. The dose covering 95% of the target (D_95%_), field size and penumbra at the center of the target were measured as a function of isocenter shift. The field size was defined as the difference in the 50% isodose lines (IDLs) on either side of the target in the direction perpendicular to the beam axis. The penumbra was defined as one half the difference between the field sizes defined by the 20% and the 80% IDLs. Field size and penumbra were measured in the longitudinal direction where the largest effect was expected due to the smaller vSAD. In the beam model utilized in this study the vSAD was 220.6 cm in the lateral direction and 183.4 cm in the longitudinal direction.

Sixteen clinical prostate patients who had been treated with proton therapy were randomly selected for the study. The cohort included 14 intact prostate and 2 prostate beds, 7 with regional LNs, and 7 with SIBs to a DIL or PET avid LN. The prescription doses to the prostate ranged from 7020 – 7920 cGy_RBE_. When pelvic LNs or intraprostatic SIBs were included, they were treated to 4500 – 5040 cGy_RBE_ and 8360 – 8800 cGy_RBE_ respectively. Two patients in the study received brachy therapy boosts. For these patients only the primary phase was delivered with PBT to 4500 – 5400 cGy_RBE_. The standard RBE value of 1.1 is used for all doses throughout this work. The plans with regional LNs do not include a PA beam. Two plans with hip prostheses were included to assess the impact on plans with oblique beams. All other plans utilized opposed lateral beams only. The clinical treatment plans were copied and re‐optimized with and without the lateral isocenter deviations in RO, holding all other optimization settings constant. The convergence criteria were such that high‐quality plans, satisfying the planning goals enforced on the clinical plan, were achievable in a single optimization cycle. The difference in single cycle optimization time was recorded. For each patient the two optimizations were run on the same server and close together in time to minimize the variability arising from workstation computational speed. The re‐optimized plans were scaled to match the V100% clinical CTV coverage and then compared in terms of dose and robustness.

A robust evaluation was carried out on all reoptimized plans following the same institutional guidelines that were applied to the clinical plans. They were evaluated in perturbation scenarios defined by ± 3.5% range uncertainty and ± 0.5 cm isocenter shifts in all directions, including the lateral direction. The robust coverage criteria V95% > 95% was required to be satisfied for all targets in all scenarios and OAR clinical goals were required to be satisfied in at least 50% of scenarios. The re‐optimized plans were also forward calculated on the clinical verification CT scans (QACTs) and required to meet the same robust coverage goal of V95% > 95%, consistent with our standard clinical procedure. All patients in the study received two QACTs during their course of treatment, one within the first three days of the start of treatment and one midway. For both the robust evaluation and the QACT analysis, the results were compared to the clinical results to identify any difference in robustness between the clinical plans and the re‐optimized plans. Statistical comparisons were carried out in Python with the stats module of the SciPy library v1.10.0, using a two‐sided paired *t*‐test. Differences were considered significant at the 95% confidence level (*p* < 0.05).^26^

## 3 RESULTS

Figure 1 shows the dose difference produced by shifting the isocenter 5 cm downstream along the beam axis for a cube field in a water phantom. The cube field is R25M4FS4 where R, M and FS indicate the depth of the distal edge of the target, and the target dimensions parallel and perpendicular to the beam axis in cm. The central axis and perpendicular dose profiles demonstrate that the beam range is unaffected while the infield dose increases and the dose in the penumbra region decreases. The effect is exactly the opposite when the isocenter is shifted upstream by the same amount. The magnitude of the effect is greatest near the surface. The dose difference perpendicular to the beam axis is asymmetric with a larger deviation in the longitudinal direction. Figure 2 shows the change in D_95%_, field size and penumbra as a function of the isocenter shift for three cube fields, R15M4FS4, R25M4FS4 and R25M4FS8. D_95%_ is expressed as a percent of the prescription dose, which was 5000 cGy_RBE_ in this study.

**FIGURE 1:**
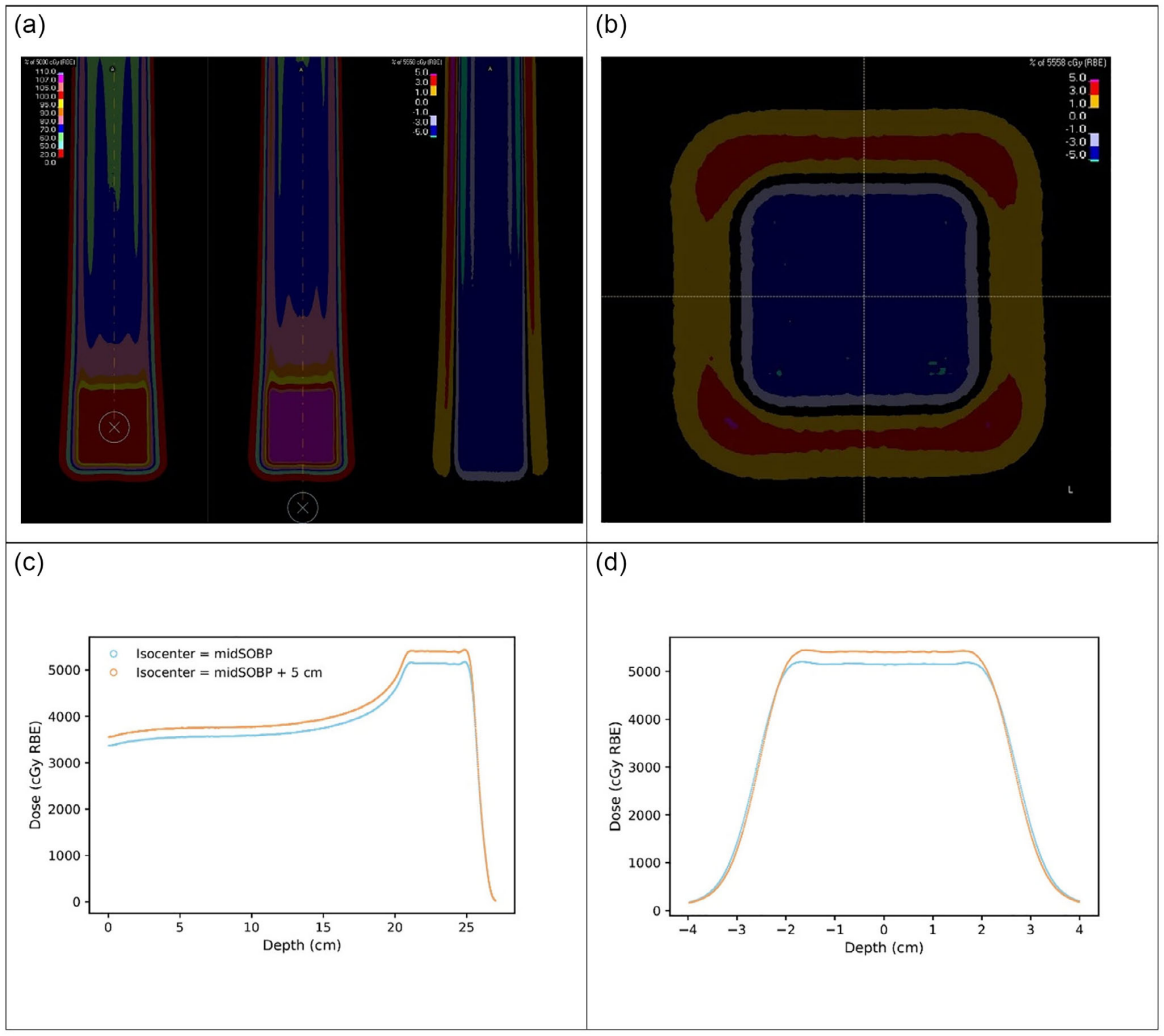
Impact of isocenter shift of a cube plan in water: (a) Comparison of the 2D dose distribution in the axial plan for the cube plan R25M10FS4 with the isocenter at the target center (left) and shifted 5 cm downstream without re‐optimization (middle) and the difference between the dose with the isocenter in the planned position and the shifted position [D_no shift_—D_5cm shift_] (right), (b) The dose difference at the center of the target in the perpendicular direction c–d) Central axis depth dose curves and perpendicular dose profiles for the nominal case with the isocenter at the target center and shifted downstream.

**FIGURE 2:**
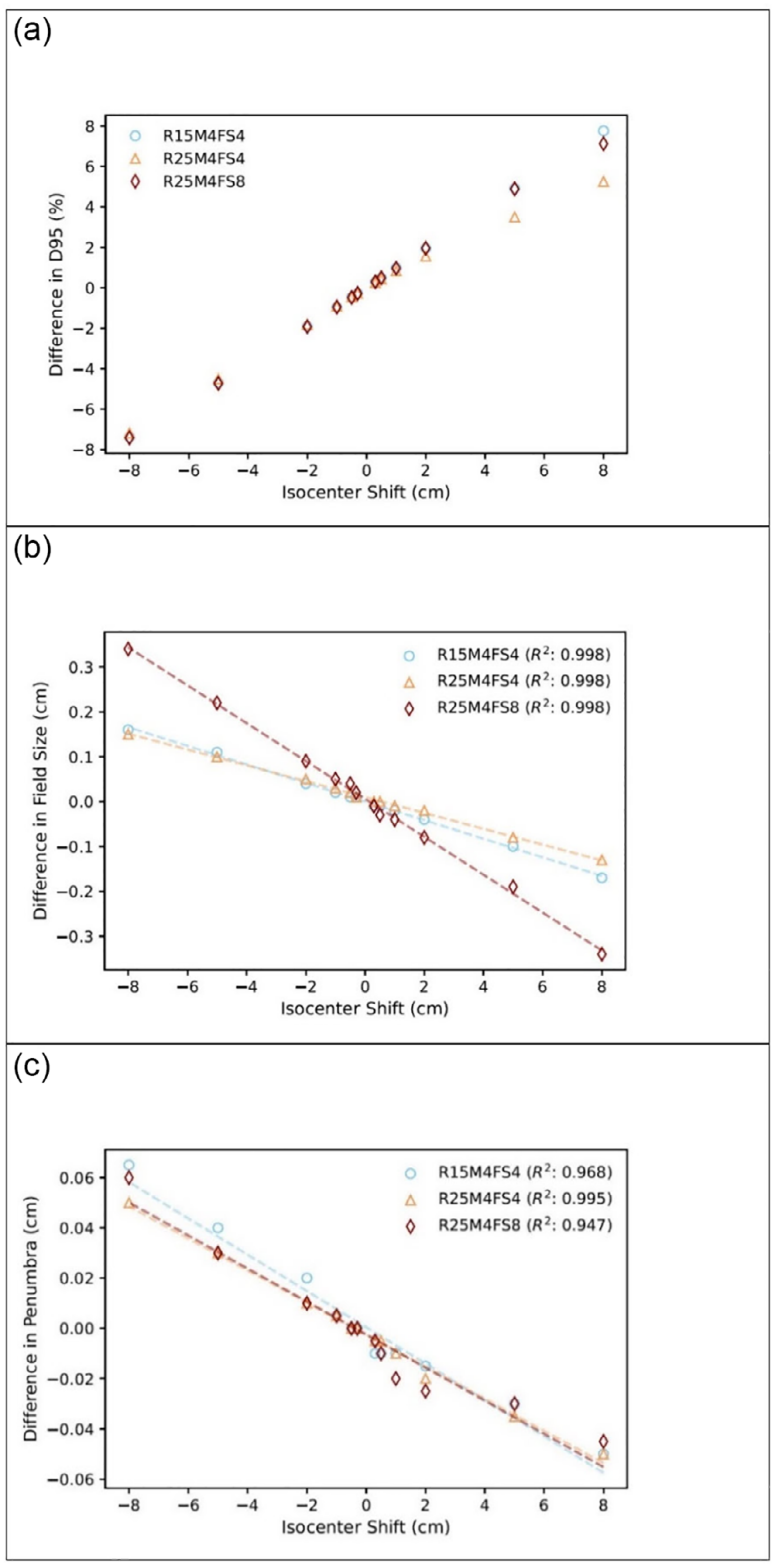
Dependence on beam range, field size and magnitude of shift: Differences in D_95%_, field size and penumbra for three cube plans as a function of isocenter shift. D_95%_ is expressed as a percent of the prescription dose, D_Rx_ = 5000 cGy RBE.

Figure 3a–d shows the impact of a similar 5 cm shift to the right in an example clinical prostate plan. Figure 4a shows a dosimetric comparison between one of the clinical prostate plans and a plan re‐optimized on the same anatomy without lateral isocenter shifts in RO, with all other optimization settings held constant. Figure 4b shows the same comparison for a plan containing oblique beams. The dosimetric and robustness comparisons between plans optimized with and without lateral isocenter shifts, are shown in Table 2 and Table 3 respectively, for all 16 patients in the study. Table 2 compares the dose‐volume‐histogram (DVH) metrics for several OARs, while Table 3 shows the results of the robust evaluation and QACT analysis. The largest difference in OAR dose was for the Bowel D_2cc_, for which the average and maximum differences were 8.92 cGy_RBE_ and 49 cGy_RBE_ respectively. The average and maximum differences in V95% were −0.01% and −0.32% for the worst scenario in RE and 0.01% and −0.05% in QACT analysis. In all cases the *p*‐values were > 0.05, which indicates no significant difference in overall plan quality, including dose and robustness. Figure 5 shows the distributions of optimization time with and without lateral shifts in RO. The average reduction in optimization time across all patients was 24 min (22%), with a *p*‐value of 0.004. The reduction ranged from a minimum of 1 min (8.3%) to a maximum of 129 min (43.1%) with a standard deviation of 32.8 min (11.3%).

**FIGURE 3:**
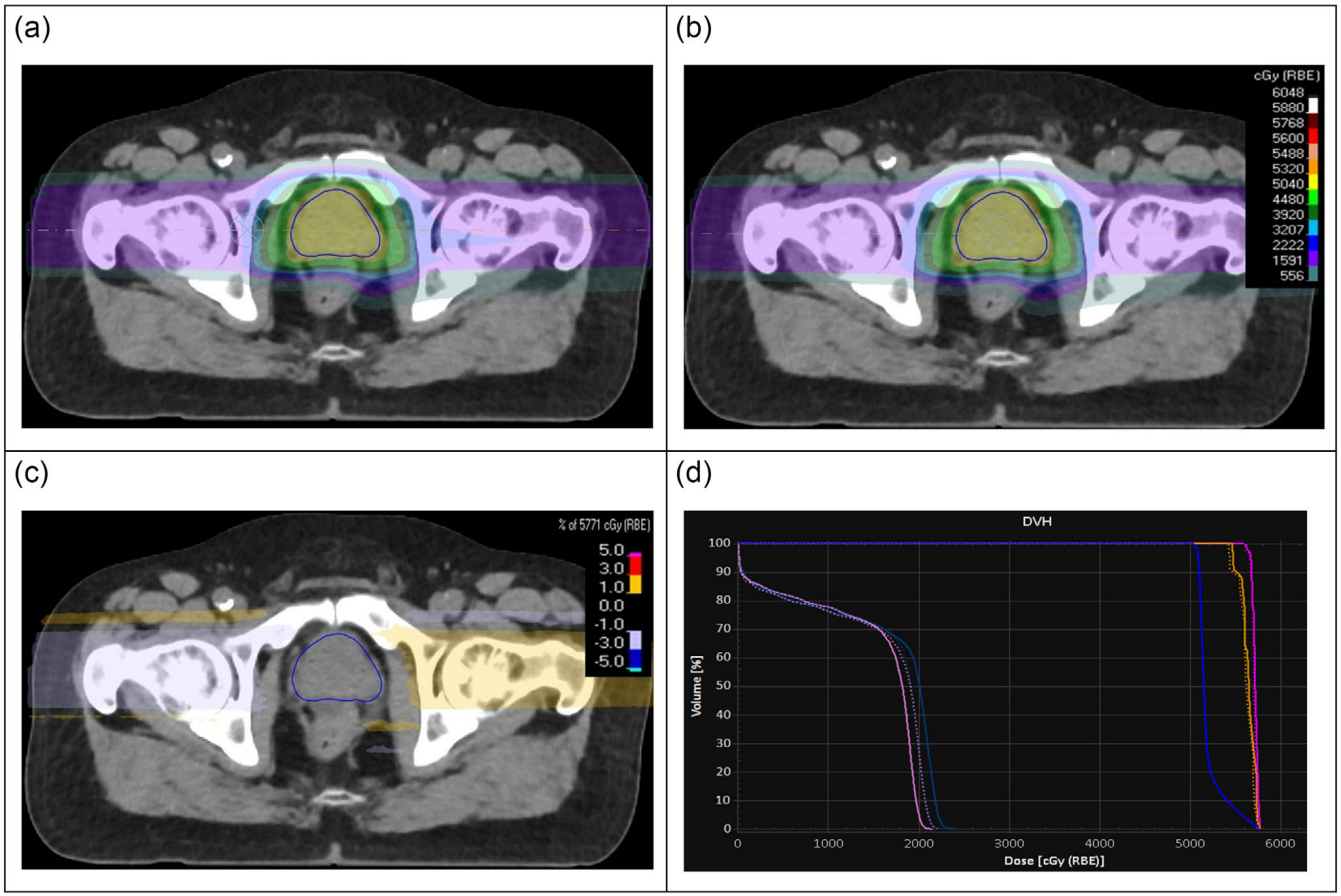
Example of impact of isocenter shift on clinical plan: (a,b) Axial dose distributions for an example clinical prostate plan with the isocenter in the planned position (top right) and shifted 5 cm to patient right (top left), (c) The dose difference (D_no shift_—D_5cm shift_), (d) DVH comparison of the planned isocenter (dotted) versus the 5 cm shift (solid).

**FIGURE 4:**
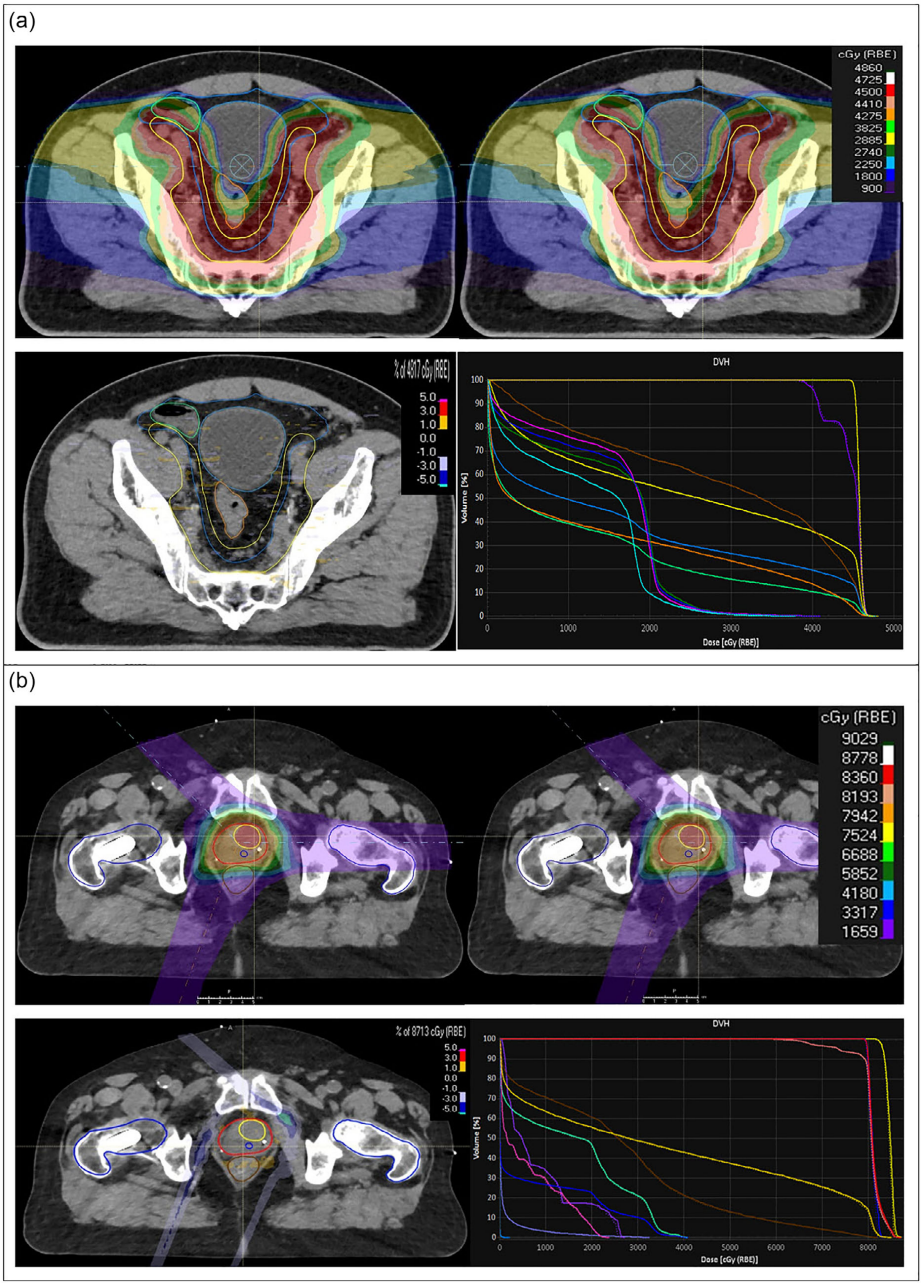
Impact of omission of lateral isocenter shifts in robust optimization: (a) Axial dose distributions for an example clinical prostate plan treated with lateral beams only, optimized with (top right) and without (top left) lateral isocenter shifts in robust optimization, the difference D_lat shift omitted_—D_lat shift included_ (bottom left) and DVH comparison (bottom right) (b) The same comparison for a clinical plan that includes an oblique beam due to the presence of a hip prosthesis. Solid DVHs represent D_lat shift omitted_ and dotted DVHs represent D_lat shift included_.

**TABLE 2:** Dosimetric comparison: OAR DVH metrics for treatment plans generated with and without lateral isocenter shifts in robust optimization. Rx indicates the dose delivered with PBT. Patients 3 and 9 received brachy therapy boosts which are not included in the data in this table. For these patients V40 is given for the bladder and rectum instead of V70. For plans with multiple target volumes treated to different dose levels only the highest dose level is shown. Doses are given in cGy RBE with a RBE value of 1.1.

		Bladder V70 (%)			Rectum V70 (%)			Sigmoid D_2cc_			Fem Head D_avg_			Bowel D_2cc_		
Patient	Rx	Shift	No Shift	Diff	Shift	No Shift	Diff	Shift	No Shift	Diff	Shift	No Shift	Diff	Shift	No Shift	Diff
1	8800	7.42	7.37	−0.05	3.93	3.91	−0.02	5566	5548	−18	2448	2442	−6	NA	NA	NA
2	8800	5.05	5.15	0.1	2.24	2.52	0.28	265	265	0	2161	2168	7	1750	1799	49
3	5040	16.92	17.12	0.2	1.92	2.05	0.13	4776	4776	0	1270	1273	3	5327	5311	−16
4	7800	0.97	0.96	−0.01	0.5	0.59	0.09	21	21	0	1643	1642	−1	17	17	0
5	7920	7.28	7.28	0	11.33	11.36	0.03	1487	1493	6	1991	1994	3	NA	NA	NA
6	7020	23.47	23.45	−0.02	14.52	14.59	0.07	4693	4705	12	2242	2241	−1	4754	4767	13
7	7920	4.14	4.16	0.02	1.47	1.49	0.02	5557	5546	−11	2565	2561	−4	5243	5249	6
8	8360	25.16	25.59	0.43	4.01	4.12	0.11	1615	1638	23	720	726	6	3207	3247	40
9	5600	40.38	40.5	0.12	19.61	20.16	0.55	5173	5194	21	1597	1598	1	5216	5218	2
10	7800	8.14	8.2	0.06	2.41	2.43	0.02	241	246	5	2163	2167	4	57	58	1
11	7920	41.71	41.87	0.16	10.94	10.92	−0.02	4673	4677	4	2459	2461	2	7128	7121	−7
12	6840	1.62	2.68	1.06	0.13	0.18	0.05	5211	5231	20	2057	2056	−1	6801	6817	16
13	8800	3.57	3.56	−0.01	1.65	1.6	−0.05	5005	5000	−5	2540	2540	0	5132	5114	−18
14	7920	9.48	9.56	0.08	4.15	4.36	0.21	5951	5954	3	1241	1242	3	7521	7553	32
15	8800	7.6	7.67	0.07	6.17	6.15	−0.02	67	68	1	2378	2382	4	NA	NA	NA
16	7920	2.53	2.49	−0.04	10.58	10.57	−0.01	2865	2898	33	1726	1726	0	2428	2426	−2
Avg:				0.14			0.09			5.88			1.25			8.92

**TABLE 3:** Robustness: Comparison of worst case robust target coverage evaluated in perturbation scenarios (± 3.5 % range, ± 0.5 cm isocenter) and on QACTs.

	Worst Case CTV V95% (%)					
	Robust Evaluation			QACT		
Patient	Shift	No Shift	Diff	Shift	No Shift	Diff
1	100	100	0	100	100	0
2	100	100	0	99.98	99.99	0.01
3	98.37	98.37	0	99.81	99.82	0.01
4	98.44	98.56	0.12	99.99	100	0.01
5	100	100	0	100	100	0
6	100	100	0	99.99	99.99	0
7	100	100	0	97.56	97.57	0.01
8	100	100	0	100	100	0
9	99.81	99.79	−0.02	99.9	99.91	0.01
10	99.86	99.86	0	100	100	0
11	100	100	0	99.94	99.96	0.02
12	93.14	93.35	0.21	98.82	99.02	0.2
13	98.26	98.22	−0.04	99.6	99.55	−0.05
14	94.18	94.14	−0.04	99.66	99.61	−0.05
15	100	100	0	100	100	0
16	95.55	95.23	−0.32	99.84	99.8	−0.04
Avg:			−0.01			0.01

**FIGURE 5:**
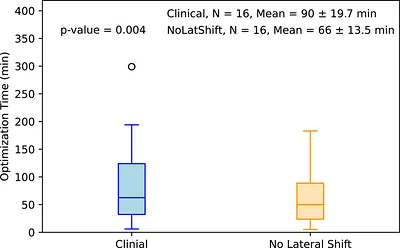
Optimization time: Box and whisker plot comparing the optimization time for plans optimized with and without lateral isocenter shifts. The boxes represent the interquartile range (IQR), and the central line represents the median of each distribution. The whiskers extend to the farthest data points that are no more than 1.5× IQR from the edges of the box.

## 4 DISCUSSION

The analysis of the cube plans in water considered very large isocenter shifts along the beam direction, far exceeding a typical clinical misalignment, to understand the underlying physical principles. It confirmed that even unrealistically large shifts have minimal impact on the beam range, which is determined primarily by the WET in the beam path. For the R25M4FS4 beam shown in Figure 1, the distal R90, defined as the depth at which the dose falls to 90% of the target mean dose beyond the target, was 25.44 cm and 25.45 cm for the nominal and shifted beams respectively. There is a visible impact on the perpendicular dose distribution. At the maximum isocenter shift of 8 cm downstream D_95%_, field size and penumbra reduce by 5.3%, 0.13 cm and 0.05 cm respectively. These deviations increase with the magnitude of the shift and field size and decrease with beam range as shown in Figure 2. These dependencies and the fact that the effect is seen more in the longitudinal direction, where the vSAD is greater, are consistent with the deviations being caused by beam divergence.

However, the deviations are negligible when considering a more clinically realistic isocenter offset.^27^ For a 0.5 cm downstream shift applied to the same cube field, the change in the same three quantities was 0.4%, 0.00 cm and −0.01 cm. The impact is reduced even further given that isocenter offsets are generally random such that they vary in size and direction from fraction to fraction, making the deviation of total plan dose less. Furthermore, the use of parallel opposed beams for prostate plans leads to a compensation effect in the target region where the dose deviation of one beam is equal and opposite to that of the other beam. As shown in Figure 3c the dose deviations of each beam effectively cancel out, making the dose in the region of the target and the principal OARs, the bladder and rectum, insensitive to isocenter shifts. There is no cancellation effect for the femoral heads, however even for the 5 cm shift shown in Figure 3, the change in average dose to the left and right femoral heads is only 50 cGy_RBE_ and −45 cGy_RBE_ respectively. For a 0.5 cm shift it is 5 cGy_RBE_ and −4cGy_RBE_, which is completely negligible. Additionally, the femoral heads are generally not a limiting constraint in prostate treatment.^28^

The comparison given in Table 2 establishes that, for the same V100% CTV coverage, there is no significant difference in the relevant DVH metrics for the main OARs of concern for any patients in the study. Perhaps more importantly, removing lateral shifts in RO did not affect the results of RE or QACT analysis. For all patients for which the clinical plan passed RE, according to the institutional criteria stipulated in the previous section, the plan optimized without lateral shifts also passed. This includes robust coverage of the prostate, LN and DIL targets. For two patients in the study the clinical plan was approved for treatment despite the robust coverage goal failing in one perturbation scenario. In these cases, the nominal coverage goal was met but robust coverage was intentionally sacrificed in favor of normal tissue sparing. In these cases, the re‐optimized plans also failed robust coverage in those scenarios. The axial dose and DVH comparison, for an example plan treating the prostate and pelvic LNs with purely lateral beams, is shown in Figure 4a. Figure 4b shows a similar comparison for a patient with a right hip replacement necessitating oblique beams on the right side. Here it is evident that the plan optimized with lateral shifts has a larger dose cloud around the edges of the oblique fields to mitigate the potential for geometric miss when the target is misaligned laterally. Despite this the DVHs agree very well, and all clinical goals are still met. The re‐optimized plan still passes RE with V95% = 100% in all scenarios and V95% = 100% for all QACTs. Although there is no negative impact of removing the lateral shifts in RO in this case involving oblique beams, it is still not recommended to omit them for cases involving oblique beams in general, since there is a visible impact in the dose distribution and the current study has not considered enough cases to conclude that it is safe to do so in general.

There is a clear clinical benefit to the reduction in optimization time that can be achieved by removing lateral isocenter shifts from RO. The distributions shown in Figure 5 are for a single cycle of optimization. A typical patient requires several cycles before a clinically acceptable plan is reached. Therefore, the actual time saved in optimization on average is likely significantly greater than 24 min. This can have a meaningful impact on clinical efficiency given the large number of prostate patients treated with proton therapy. The optimization times shown in Figure 5 and the differences given in the results section are subject to uncertainty associated with the speed of the server that the calculation was carried out on, which varies in a random way between servers and over time. This cannot be controlled directly since the user cannot control what server will be selected nor the computational load of that server from other processes at a given time. It is desirable that this uncertainty was included in the study since it mirrors the uncertainty in optimization time seen by clinical planners. Despite this a clear pattern emerges showing a reduction in optimization time in all plans. Furthermore, although the optimization times themselves are subject to this uncertainty, the degree to which this impacted the difference in optimization time was limited by running both optimizations for each patient on the same server and as close together in time as possible.

## 5 CONCLUSION

For prostate cancer patients treated with PBS proton therapy utilizing bilateral opposed fields, lateral isocenter shifts can be safely omitted from robust optimization to minimize optimization time and increase overall clinical efficiency, with no negative impact on plan quality. It is not recommended to omit them in cases with non‐lateral beams, such as plans requiring oblique beams to avoid hip prostheses or posterior beams to help cover lymph nodes. On the basis of these results our institutional policy was modified to no longer require lateral shifts in robust optimization in these cases. The time and resources saved can allow for an increase in patient throughput or can be redirected to other activities that improve clinical practice. Increasing patient throughput is essential for proton therapy centers given the limited access to this advanced treatment modality. Increasing efficiency in treatment planning can extend the benefits of proton therapy to more patients.

## AUTHOR CONTRIBUTIONS

**Fionnbarr O'Grady**: Conceptualization; methodology; data curation and analysis; writing original draft. **Jackeline Castro**: Conceptualization; methodology; validation. **Daniel W Kim**: Methodology and validation. **Jiajin Fan**: Methodology and validation. **Peng Wang**: Methodology and validation.

## CONFLICT OF INTEREST STATEMENT

The authors have no relevant conflicts of interest to disclose.

## ETHICS AND IRB COMPLIANCE

This work has been approved by our institutional IRB.

## Data Availability

Data is available from the corresponding author upon request.
